# Two Cases of Posner-Schlossman Syndrome With the Detection of Human Herpesvirus 6 DNA in the Aqueous Humor

**DOI:** 10.7759/cureus.113564

**Published:** 2026-07-29

**Authors:** Atsuki Fukushima, Hiroyuki Wakuda, Hitoshi Tabuchi

**Affiliations:** 1 Ophthalmology, Tsukazaki Hospital, Himeji, JPN; 2 Ophthalmology, Hiroshima University, Hiroshima, JPN

**Keywords:** aqueous humor, cytomegalovirus, human herpesvirus 6, polymerase chain reaction, posner-schlossman syndrome

## Abstract

Posner-Schlossman syndrome (PSS) is one of the disorders characterized by the acute elevation of intraocular pressure (IOP). Because cytomegalovirus (CMV), varicella-zoster virus (VZV), and herpes simplex virus (HSV) have been detected in the aqueous humor of patients with PSS, a viral etiology has long been suspected. We encountered two patients with PSS in whom a polymerase chain reaction (PCR) analysis of aqueous humor detected human herpesvirus 6 (HHV-6) DNA. Both patients presented with elevated IOP and anterior chamber inflammation. Treatment with topical betamethasone and topical ganciclovir resulted in the normalization of IOP and the resolution of anterior chamber inflammation. Previous reports have suggested an association between uveitis and HHV-6 infection. Therefore, HHV-6 may also play a role in the pathogenesis of Posner-Schlossman syndrome.

## Introduction

Posner-Schlossman syndrome (PSS) is a disorder characterized by recurrent episodes of an acute elevation of intraocular pressure (IOP) together with mild nongranulomatous anterior uveitis. In 1948, Posner and Schlossman first described nine cases of a distinctive form of glaucoma characterized by unilateral acute ocular hypertension despite an open anterior chamber angle [1]. This constellation of clinical findings subsequently became known as Posner-Schlossman syndrome [2]. The characteristic features of PSS are recurrent episodes of mild nongranulomatous anterior uveitis accompanied by the unilateral elevation of intraocular pressure [2,3].

Although the exact etiology of PSS remains unclear, an infectious mechanism is now widely accepted. Cytomegalovirus (CMV), varicella-zoster virus (VZV), and herpes simplex virus (HSV) have all been detected in the aqueous humor of affected patients [4]. It is possible that other viral infections play a role in the development of PSS, and identifying another specific viral etiology will be important for specific antiviral treatment, recurrence prevention, and glaucoma-related complications.

Human herpesvirus 6 (HHV-6) is a well-known causative agent of exanthem subitum (roseola infantum) in children [5]. In ophthalmology, HHV-6 has been reported to cause uveitis, particularly in immunocompromised patients [6-8]. HHV-6 has also been implicated in corneal endotheliitis in association with CMV and HSV-1 infections [9,10]. However, no previous reports have described an association between HHV-6 and Posner-Schlossman syndrome.

Here, we report two cases of PSS in which HHV-6 DNA was detected in aqueous humor by polymerase chain reaction (PCR). To the best of our knowledge, this is the first report suggesting that HHV-6 infection may be present in patients diagnosed with Posner-Schlossman syndrome.

## Case presentation

Case 1

A 60-year-old man with no significant medical history presented to a local ophthalmologist with a one-week history of blurred vision and ocular pain in his right eye as the first episode. His intraocular pressure (IOP) was 36 mmHg in the right eye, and dorzolamide hydrochloride/timolol maleate was prescribed. The next day, he was referred to our department for further evaluation.

At the initial examination, IOP was 22 mmHg in the right eye and 16 mmHg in the left eye. Best-corrected visual acuity was 20/13 in the right eye and 20/17 in the left eye.

**Figure 1:**
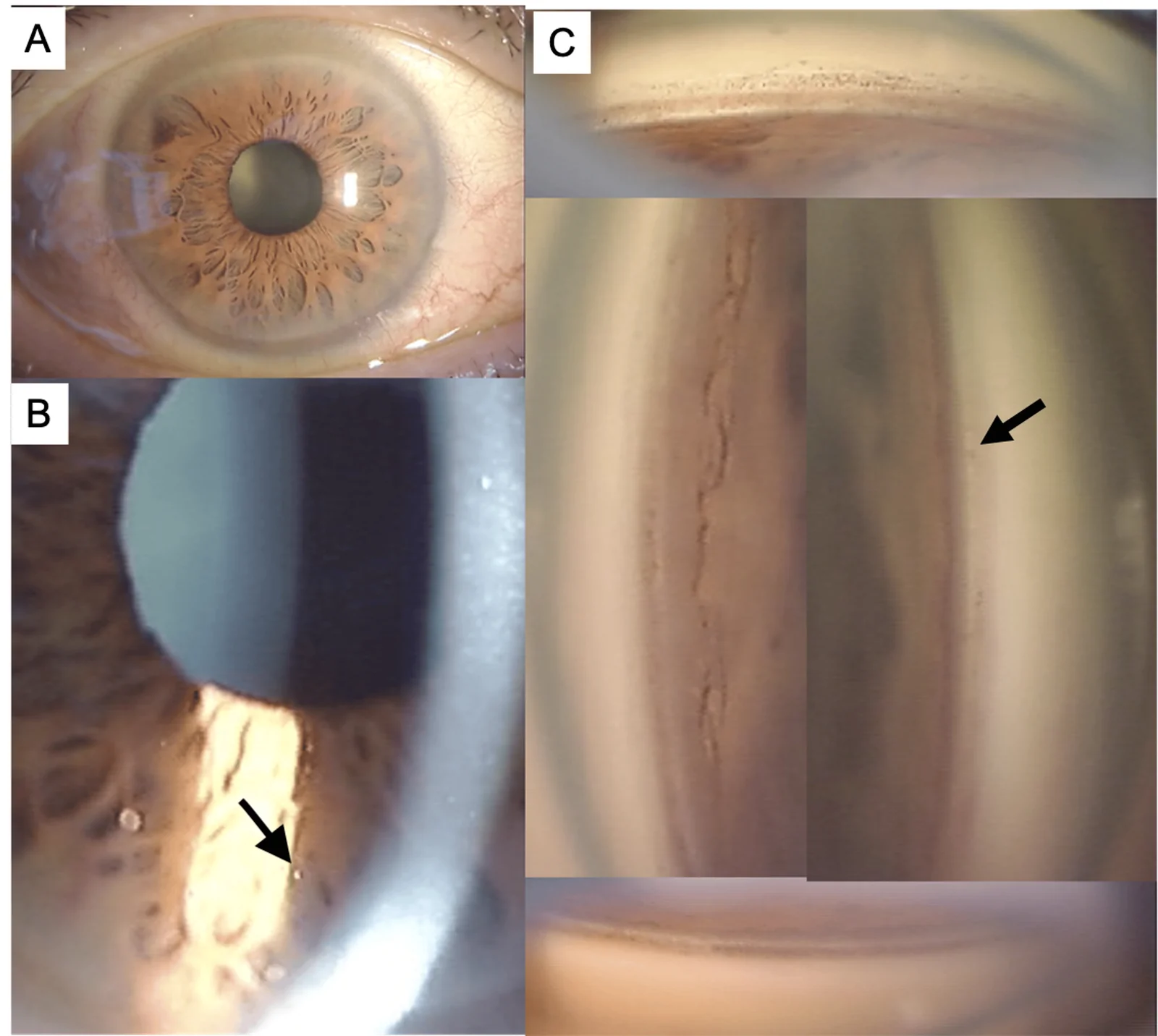
Slit-lamp examination of Case 1. Mild iris atrophy (A) and fine keratic precipitates (B, arrow) are observed in the right eye. Gonioscopy demonstrated open anterior chamber angles with slightly reduced trabecular meshwork pigmentation in the right eye (C, arrow). No peripheral anterior synechiae were observed in the right eye (C).

Slit-lamp examination revealed mild iris atrophy (Figure 1A), fine keratic precipitates (Figure 1B), and 1+ anterior chamber cells in the right eye, followed by grading ocular inflammation and uveitis with the Standardization of Uveitis Nomenclature (SUN) Criteria. No inflammatory findings were observed in the left eye. Gonioscopy demonstrated open anterior chamber angles in both eyes, with slightly reduced trabecular meshwork pigmentation in the right eye (Figure 1C). No peripheral anterior synechiae were observed (Figure 1C, Scheie’s system of grading angle pigmentation: I).

Laser flare photometry showed flare values of 14.2 photon counts/ms in the right eye and 4.6 photon counts/ms in the left eye (Kowa FM-600α, Tokyo, Japan; normal range: 5-10 photon counts/ms). Fundus examination and both anterior-segment (AS) and retinal nerve fiber layer (RNFL) optical coherence tomography (OCT) revealed no remarkable abnormalities.

One hundred microliters of aqueous humor was collected using a 27 G needle and subjected to multiplex PCR analysis at the initial visit. Human herpesvirus 6 (HHV-6) DNA was detected. However, the PCR analysis of aqueous humor was negative for herpes simplex virus type 1 (HSV-1), herpes simplex virus type 2, varicella-zoster virus, Epstein-Barr virus, cytomegalovirus, human T-cell leukemia virus, *Treponema pallidum*, and *Toxoplasma gondii*. Therefore, we considered that the ocular inflammation may be associated with HHV-6 infection.

The ocular inflammation was therefore considered to be associated with HHV-6 infection. Topical 1% ganciclovir was initiated six times daily in the right eye. One week after treatment, IOP had decreased to 10 mmHg in the right eye and 12 mmHg in the left eye, while flare values decreased to 7.5 and 4.5 photon counts/ms, respectively. The anterior chamber inflammation also improved.

The frequency of topical ganciclovir was gradually reduced to four times daily. One month later, IOP remained within the normal range (7 mmHg in the right eye and 9 mmHg in the left eye), and flare values further decreased to 4.2 and 6.4 photon counts/ms, respectively. Medication was continued for six months, and no recurrence has been observed for two years.

Case 2

A 41-year-old man noticed blurred vision in his left eye in the morning and visited an eye clinic. Anterior uveitis was found, and on the same day, he was referred to our department. He had no significant past medical history.

At presentation, intraocular pressure (IOP) was 10 mmHg in the right eye and 34 mmHg in the left eye. Best-corrected visual acuity was 20/13 in the right eye and 20/20 in the left eye.

**Figure 2:**
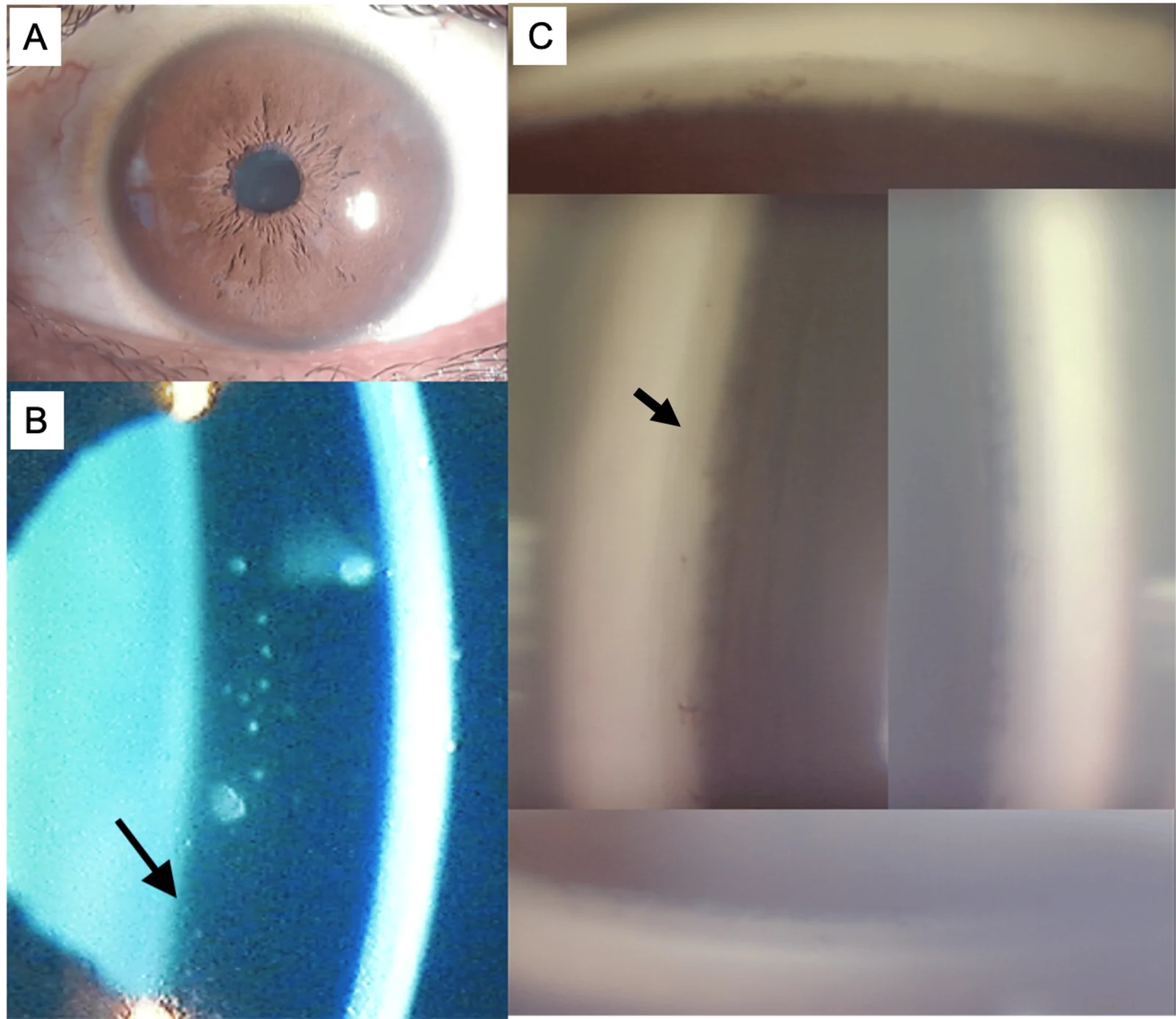
Slit-lamp examination of Case 2. Iris atrophy was not observed (A), while fine keratic precipitates (B, arrows) were noted in the left eye. Gonioscopy demonstrated open anterior chamber angles with minimal trabecular meshwork pigmentation in the left eye (C, arrow). No peripheral anterior synechiae were present in the left eye (C).

Slit-lamp examination revealed that iris atrophy was not observed (Figure 2A), while fine keratic precipitates (Figure 2B) and 1+ anterior chamber cells were noted in the left eye. Gonioscopy demonstrated open anterior chamber angles in both eyes, with minimal trabecular meshwork pigmentation bilaterally (Figure 2C, Scheie’s system of grading angle pigmentation: none). No peripheral anterior synechiae were present (Figure 2C).

Laser flare photometry showed flare values of 5.0 photon counts/ms in the right eye and 33.2 photon counts/ms in the left eye (Kowa FM-600α; normal range: 5-10 photon counts/ms). Fundus examination and both AS and RNFL OCT revealed no remarkable abnormalities.

The patient was diagnosed with Posner-Schlossman syndrome presumably and was treated with topical betamethasone six times daily and topical tropicamide/phenylephrine (Mydrin®) once daily. One day after the initiation of treatment, the IOP decreased to 15 mmHg.

One hundred microliters of aqueous humor was collected using a 27 G needle and subjected to multiplex PCR analysis at the initial visit. Human herpesvirus 6 (HHV-6) DNA was detected. PCR results were negative for herpes simplex virus type 1, herpes simplex virus type 2, varicella-zoster virus, Epstein-Barr virus, cytomegalovirus, human T-cell leukemia virus, *Treponema pallidum*, and *Toxoplasma gondii*. No significant abnormalities were detected on serological examination.

Therefore, we considered that the ocular inflammation may be associated with HHV-6 infection. Topical 1% ganciclovir was initiated six times daily, and topical betamethasone was gradually tapered. After two weeks of treatment, laser flare photometry had normalized to 7.5 photon counts/ms. Medication was continued for four months, and no recurrence has been observed for two years.

## Discussion

We encountered two cases of presumed Posner-Schlossman syndrome (PSS) in which human herpesvirus 6 (HHV-6) was detected. In the first case, HHV-6 infection was diagnosed at an early stage, and treatment with topical ganciclovir was initiated immediately. In the second case, treatment was initially started with topical betamethasone, resulting in the improvement of inflammation within one week. However, subsequent polymerase chain reaction (PCR) testing suggested the involvement of HHV-6. In both cases, topical ganciclovir therapy successfully resolved the ocular inflammation and normalized intraocular pressure (IOP). To the best of our knowledge, the presence of HHV-6 in patients diagnosed with presumed PSS has not previously been reported.

In 1948, Posner and Schlossman described a disorder characterized by recurrent elevations of IOP and termed it glaucomatocyclitic crises [1], which later became known as Posner-Schlossman syndrome [2]. The clinical characteristics of PSS remain largely consistent with the original description: an open anterior chamber angle, recurrent unilateral elevations of IOP, mild anterior chamber inflammation, small-to-medium-sized keratic precipitates, mild conjunctival hyperemia, and the absence of posterior synechiae [4]. Both of our cases fulfilled these diagnostic features except for recurrent IOP elevation, as topical treatment was effective and the follow-up period was relatively short, and therefore, we considered the diagnosis of these cases as PSS presumably.

HHV-6 has been reported as a possible cause of uveitis and corneal endotheliitis. de Groot-Mijnes et al. reported the detection of HHV-6 in the aqueous humor of one patient among 139 cases of idiopathic uveitis in which herpes simplex virus, varicella-zoster virus, cytomegalovirus, and *Toxoplasma gondii* had all tested negative by PCR [8]. Glâtre et al. reported HHV-6-associated unilateral uveitis in a 47-year-old patient receiving etanercept therapy for rheumatoid arthritis [7]. Maslin et al. reported the detection of HHV-6 in a patient with bilateral uveitis [6]. Sugita et al. performed a multiplex PCR analysis of intraocular fluid samples from 100 patients and detected a high copy number of HHV-6 in one patient with severe unilateral uveitis [9]. In addition, Ono et al. [11] reported a case in which the reactivation of HHV-6 was associated with herpes simplex virus type 1 (HSV-1) corneal endotheliitis, and Yokogawa et al. [12] described a case of corneal endotheliitis with concurrent cytomegalovirus and HHV-6 infection. Collectively, these reports suggest that HHV-6 may contribute to ocular inflammation either as a sole pathogen or in conjunction with other viruses.

Among these previous reports, elevated IOP was documented only in the cases reported by Ono et al. [11] and Yokogawa et al. [12], both of which involved co-infection with HHV-6 and another virus. To the best of our knowledge, no cases of elevated IOP associated with HHV-6 infection alone have been reported.

At present, there is no established consensus regarding the treatment of HHV-6 ocular infection. However, the efficacy of ganciclovir [6,13] and foscarnet [13] has been suggested. In the present cases, topical ganciclovir was effective in both patients, suggesting the possibility that HHV-6 played a key role in the pathogenesis. Neither patient has experienced recurrence to date, although careful long-term follow-up is warranted.

Although HHV-6 was detected by PCR in both cases, this finding alone does not establish HHV-6 as the underlying cause of PSS. Because HHV-6 infection is nearly universal during childhood, the detection of viral DNA in aqueous humor may simply reflect the presence of latent virus rather than active pathogenic infection. In the present study, only a single PCR assay was performed, and viral load could not be quantified. Therefore, asymptomatic latent HHV-6 infection cannot be excluded. To establish HHV-6 as the causative pathogen, serial quantitative real-time PCR analyses and serological evaluation, including changes in HHV-6-specific IgM antibody titers, would be necessary.

## Conclusions

We encountered two patients with presumed PSS in whom a PCR analysis of aqueous humor detected HHV-6 DNA. Treatment with topical ganciclovir together with either intraocular pressure‑lowering or betamethasone drops resulted in the normalization of IOP and the resolution of anterior chamber inflammation. Taken together, HHV-6 may also play a role in the pathogenesis of PSS. Further studies are necessary to confirm that HHV-6 is one of the causes of PSS.
